# Heparin-binding protein and procalcitonin in the diagnosis of pathogens causing community-acquired pneumonia in adult patients: a retrospective study

**DOI:** 10.7717/peerj.11056

**Published:** 2021-03-12

**Authors:** Rentian Cai, Huihui Li, Zhen Tao

**Affiliations:** 1 Department of Infectious Disease, Nanjing First Hospital, Nanjing Medical University, Nanjing, China; 2 Department of Infectious Disease, Nanjing Medical University, Nanjing, China

**Keywords:** Heparin-binding protein, Procalcitonin, C-reactive protein, Community acquired pneumonia

## Abstract

The performance of inflammatory markers in community-acquired pneumonia (CAP) caused by different pathogens has not been fully studied. We sought to find the differences in the concentrations of procalcitonin (PCT) and heparin-binding protein (HBP) between patients with CAP caused by different pathogens. We enrolled 162 patients with CAP, divided into three groups on the basis of bacterial (*n* = 108), fungal (*n* = 21) and viral (*n* = 33) infection. Complete leukocyte counts and the concentration of HBP and PCT were measured, and the differences were compared with nonparametric tests. The receiver operating characteristic (ROC) curve was used to evaluate the significant differences in the sensitivity and specificity of the indicators. The leukocyte and neutrophils counts and the concentrations of HBP and PCT in the viral group were significantly lower than those in the other two groups (*p* < 0.001). The area under the ROC curve (AUC) of the concentration of HBP and PCT as well as leukocyte and neutrophils counts were 0.927, 0.892, 0.832 and 0.806 for distinguishing bacterial from viral infection, respectively. The best cut-off value was 20.05 ng/mL for HBP, with a sensitivity of 0.861 and specificity of 0.939. The best cut-off value was 0.195 ng/mL for PCT, with a sensitivity of 0.991 and specificity of 0.636. The best cut-off value was 5.195 × 10^9^/L and 4.000 × 10^9^/L for leukocyte and neutrophils counts, with sensitivity of 0.694 and 0.880 and specificity of 0.667 and 0.636, respectively. The AUC of HBP, PCT and leukocyte and neutrophil counts for distinguishing fungal from viral infection were 0.851, 0.883, 0.835 and 0.830, respectively. The best cut-off values were 29.950 ng/mL, 0.560 ng/mL, 5.265 × 10^9^/L and 3.850 × 10^9^/L, with sensitivity of 0.667, 0.714, 0.905 and 0.952 and specificity of 0.970, 0.879 0.667 and 0.606, respectively. There were no significant differences in the three indicators between the bacterial and fungal infection groups. The concentration of CRP showed no significant differences among the three groups. Consequently, the stronger immune response characterized by higher inflammation markers including HBP and PCT can help distinguish bacterial and fungal CAP from viral CAP.

## Introduction

Infectious diseases are most frequently seen in clinical practice (Rudd et al., 2020), and community-acquired pneumonia (CAP) is by far the most common among them (Armitage & Woodhead, 2007; GBD 2016 Causes of Death Collaborators, 2017; Wunderink & Waterer, 2017).

The most common pathogens are bacteria, fungi and virus, which can be identified according to sputum culture, second-generation sequencing and other methods. It is important to identify the pathogenic microorganisms to choose the appropriate therapeutic regimen. However, cultivation and identification of bacteria, fungi and viruses are time-consuming (Collins, Popowitch & Miller, 2020). Although second-generation sequencing plays a complementary role in the identification of pathogenic organisms, it is expensive and not the preferred method for the diagnosis of infectious diseases in developing countries. In fact, different pathogens can induce different types of inflammation (Atri, Guerfali & Laouini, 2018; Murdoch & Finn, 2000). C-reactive protein (CRP), procalcitonin (PCT) and heparin-binding protein (HBP) are commonly used in clinical practice to evaluate inflammatory response (Dolin et al., 2018; Larsen & Petersen, 2017; Linder et al., 2012; Sproston & Ashworth, 2018; Zhang et al., 2019). They are used to evaluate the therapeutic effect on infectious diseases, but they have not been fully explored for identifying the species of pathogens.

CRP is considered to be an early indicator of the acute phase of infection and inflammation (Chen, Fei & Deirmegian, 2014; Kizer et al., 2011; Meidani et al., 2013). It is synthesized by hepatocytes and activates, complements and promotes the phagocytosis of granulocytes and macrophages (Dandona et al., 1994). PCT, a precursor of calcitonin, is secreted mainly by parathyroid C cells. PCT is an inflammation-related serological marker widely used in clinical practice (Becker, Snider & Nylen, 2010), and is secreted by monocytes and macrophages in the infective stage (Linscheid et al., 2004; Zheng et al., 2018). PCT plays a pivotal role in the diagnosis of sepsis (Beqja-Lika et al., 2013; Schlattmann & Brunkhorst, 2014; Wacker et al., 2013). HBP is a granular protein secreted by neutrophils, which has significant sterilization activity, chemotaxis and inflammatory regulation (Fisher & Linder, 2017; Shafer, Martin & Spitznagel, 1984). HBP can be used as a marker for bacterial infection (Linder et al., 2012; Linder, Soehnlein & Akesson, 2010). However, the ability of these inflammatory markers to identify and distinguish the bacteria, fungi and viruses causing CAP has rarely been studied.

Therefore, we sought to determine the differences in concentrations of PCT and HBP in patients with CAP caused by different pathogens, to assist with the choice of appropriate treatment.

## Materials and Methods

### Study population and data collection

The patients’ data were collected in Nanjing First Hospital from September 1, 2018 to May 31, 2019. The inclusion criteria included: (1) age >18 years; (2) onset in the community; (3) new patchy infiltrates, lobar or segmental consolidation, ground-glass opacities or interstitial changes with or without pleural effusions; (4) new onset of cough or expectoration, or aggravation of existing symptoms of respiratory diseases, with or without purulent sputum, chest pain, dyspnea or hemoptysis or signs of pulmonary consolidation and/or moist rales (Cao et al., 2018); and (5) all cases were confirmed by viral nucleic acid assays with real-time polymerase chain reaction (PCR) including influenza A virus, influenza B virus, human parainfluenza virus, adenovirus and respiratory syncytial virus, using nasopharyngeal swab and sputum smear and culture. Exclusion criteria: patients with human immunodeficiency virus (HIV), hepatitis B virus (HBV), hepatitis C virus (HCV) and tuberculosis infection; autoimmune diseases; long-term corticosteroid use for >6 months. We enrolled 162 patients. Demographic patient data, including age, sex and infection sites, were obtained from medical records. The computed tomography (CT) score was used to quantitatively estimate the pulmonary involvement in all these abnormalities on the basis of the area involved (Chang et al., 2005; Pan et al., 2020). Each of the five lung lobes were visually scored from 0 to 5 as: 0, no involvement; 1, <5% involvement; 2, 25% involvement; 3, 26%–49% involvement; 4, 50%–75% involvement; 5, >75% involvement. The total CT score was the sum of the individual lobar scores and ranged from 0 (no involvement) to 25 (maximum involvement). Confusion, uremia, elevated respiratory rate, hypotension, and aged 65 years or older (CURB-65) score was enrolled.

According to the clinical manifestations, sputum culture and PCR results, the patients were divided into three groups: bacterial, fungal and viral infection groups (Tables 1 and 2).

### Ethical approval and consent to participate

The study protocols were approved by the Research Ethics Committee of Nanjing First Hospital. The need for written informed consent from the participants was waived by the committee because our study was retrospective, anonymous and only used currently existing data.

### Investigations

We used EDTA, heparin and citrate for assays of CRP, PCT and HBP, respectively. Blood samples were taken within 2 h after admission of patients. Complete leukocyte counts, liver function tests including serum concentration of alanine aminotransferase (ALT) and total bilirubin (TB), as well as renal function tests including blood urea nitrogen (BUN) and creatinine (Cr) were measured as described in our previous research (Cai et al., 2017). The plasma concentration of CRP was tested within 2 h by turbidimetric inhibition immunoassay, normal range: 0–8 mg/L (PA900, Lifotronic, China). The plasma concentration of PCT was measured using an automatic chemiluminescence immunoassay analyzer within 2 h, normal range: 0–0.1 ng/mL (cobas e 601, Roche, Switzerland). The detection of HBP concentration in plasma was detected within 2 h using the Axis-Shield HBP microtiter plate ELISA kits using sodium citrate tubes with the automatic rapid immunoassay system, normal range: 0–11.4 ng/mL (Jet-iStar 3000, Joinstar, China).

**Table 1:** General characteristics of the study subjects.

Variable	Bacteria(*n* = 108)	Fungus(*n* = 21)	Virus(*n* = 33)	*p*
Sex (% male)	71(65.74)	12(57.14)	19(57.58)	0.139
Age (mean ± s, year)	69.93 ± 17.28	67.76 ± 17.78	64.79 ± 21.10	0.348
Mortality (n, %)	1(0.93)	0(0)	1(3.03)	0.557
Complications				
Heart disease	39	8	13	0.938
Stroke	22	5	5	0.710
COPD	20	4	5	0.897
Hypertension	50	7	10	0.191
Type 2 diabetes	19	6	5	0.424
Tumor	10	5	0	0.012
Kidney disease	3	0	0	1.000
CURB-65 score	2(1, 2)	2(1, 2)	1(1, 2)	0.550
HBP(ng/mL)	48.85(33.57, 78.54)	52.90(8.51, 126.00)	11.05(8.01, 16.12)	<0.001
CRP(mg/L)	73.00(20.13, 125.00)	52.90(8.51, 126.00)	40.60(10.75, 100.20)	0.158
PCT(ng/mL)	1.00(0.50, 2.45)	0.80(0.45, 4.00)	0.17(0.06, 0.43)	<0.001
Leukocyte(×10^9^/L)	8.99(6.45, 13.24)	9.38(5.78, 14.62)	3.89(2.13, 7.64)	<0.001
Neutrophils(%)	80.20(71.43, 86.78)	81.00(71.35, 86.90)	81.40(70.15, 85.75)	0.874
Neutrophils(×10^9^/L)	6.90(4.90, 11.15)	7.50(4.33, 10.95)	2.6(1.55, 6.10)	<0.001
Monocyte(%)	6.15(3.53, 8.38)	5.10(3.60, 7.15)	4.40(2.90, 7.40)	0.118
Lymphocyte(%)	11.15(7.63, 18.98)	10.70(5.95, 16.70)	12.00(6.10, 20.45)	0.882
ALT(U/L)	19.75(12.03, 34.48)	24.30(14.00, 27.65)	22.10(16.55, 39.50)	0.126
TB(µmol/L)	8.60(6.10, 12.68)	9.10(4.60, 12.90)	8.70(6.00, 14.30)	0.733
BUN(mmol/L)	8.90(7.03, 11.93)	8.00(7.15, 11.90)	7.90(6.70, 9.35)	0.165
Cr(µmol/L)	67.55(56.33, 90.80)	73.00(48.95, 117.00)	69.60(54.05, 86.15)	0.575
CT-score	3(2, 5)	3(2, 4)	4(3, 5)	0.081
PaO2/FiO2	338.61 ± 52.15	334.04 ± 47.78	342.42 ± 47.73	0.838

**Notes.**

COPD chronic obstructive pulmonary disease HBP heparin binding protein CRP c-reactive protein PCT procalcitonin ALT alanine aminotransferase TB total bilirubin BUN blood urea nitrogen Cr creatinine

**Table 2:** Different pathogens of three kinds of pneumonia.

Type of pneumonia	pathogens	numbers of patients
Bacterial pneumonia	Streptococcus pneumoniae	2
	Staphylococcus aureus	2
	Escherichia Coli	1
	Haemophilus influenzae	1
	Proteus mirabilis	1
	Maltophilia Stenotrophomonas	1
	Serratia marcescens	1
	Enterobacter cloacae	3
	Acinetobacter baumannii	8
	Pseudomonas aeruginosa	8
	Klebsiella pneumoniae	11
	Culture negative, but smear Gram-negative bacilli	16
	Culture negative, but smear Gram-positive cocci	53
Fungal pneumonia	Aspergillus	21
Viral pneumonia	Influenza A virus	27
	Influenza B virus	1
	Human parainfluenza virus	1
	Adenovirus	3
	Respiratory syncytial virus	1

### Statistical methods

The variance homogeneity of the data was evaluated by Levene’s test. The Shapiro–Wilk test was used to assess data normality. Normally and non-normally distributed data were shown as mean ± standard deviation and median and interquartile range (IQR), respectively. The differences in measurement data were compared with Student’s *t*-test if the data met normal distribution and homogeneity of variance, or with a non-parametric test. The Mann–Whitney U test was used to compare the statistical significance between the groups with non-normally distributed data. The enumeration data were compared with the chi-squared test. The receiver operating characteristic (ROC) curve was used to evaluate the significant differences of the sensitivity and specificity of indicators. The results were considered significant when the *p*-values were ≤0.05. All statistical analyses were conducted using GraphPad Prism 6.0 (San Diego, CA, USA) and IBM SPSS version 22 (IBM SPSS, Armonk, NY, USA).

## Results

### Characteristics of the study population

The clinical characteristics of the 162 patients enrolled are shown in Table 1. There were no significant differences in the age and male proportion among the three groups. The proportion of tumors in the fungal group was significantly higher (5/21, 23.81%) than that in the viral group (0%), and there were no significant differences in the proportion of complications among the three groups. The CURB-65 score, CT score, PaO_2_/FiO_2_, liver function and kidney function showed no significant differences among the three groups (Table 1). The different pathogens are shown in Table 2.

### Accessible blood index

#### HBP

**Figure 1:**
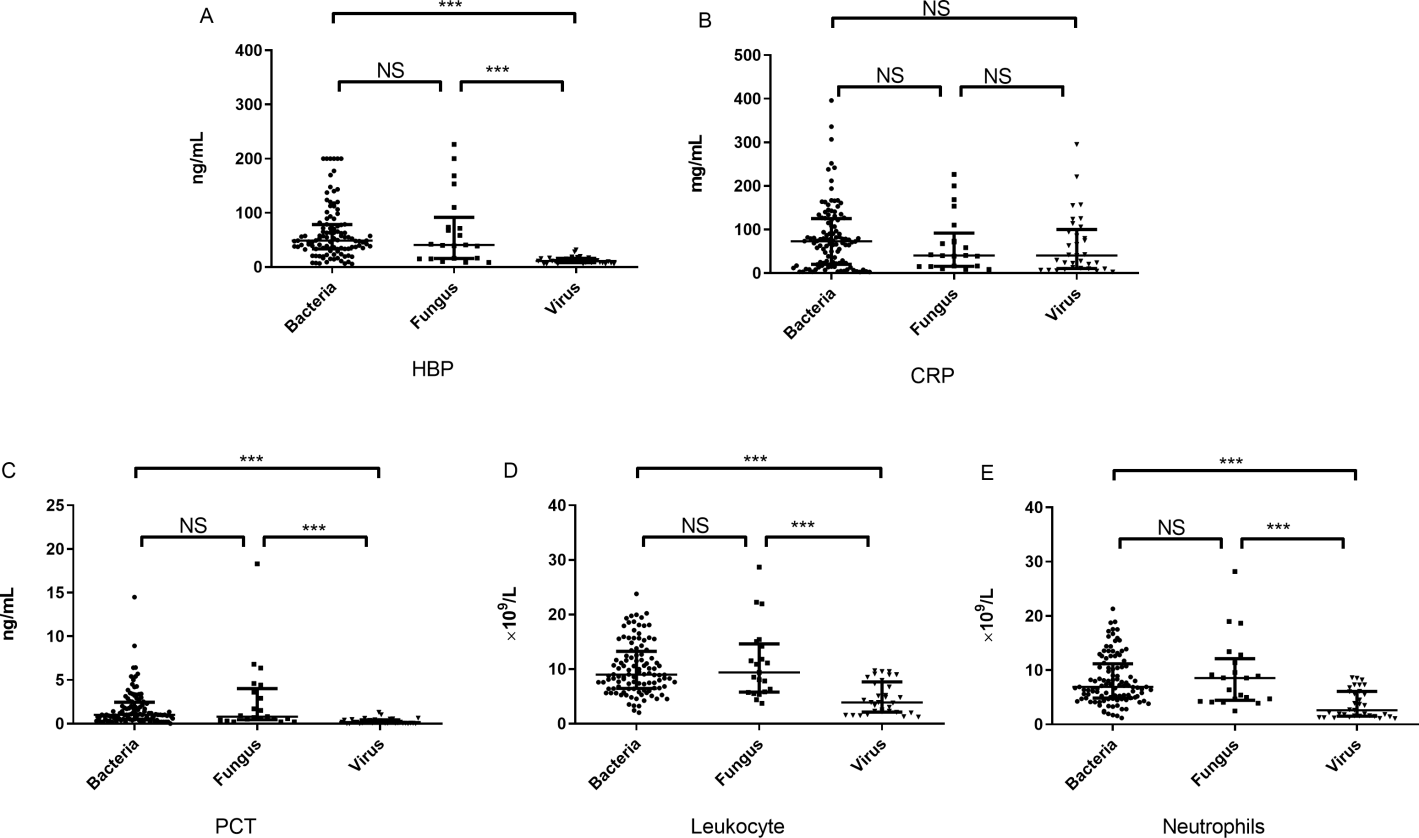
The concentration of inflammatory markers in peripheral plasma of three groups. The concentrations of HBP (A), CRP (B), PCT (C) as well as the counts of leukocyte (D) and neutrophils (E) in the bacterial, fungal and viral groups. note: HBP, heparin-binding protein; CRP, C-reactive protein; PCT, procalcitonin; NS, no significance; ***, *P* < 0.001.

There were significant differences in the concentration of HBP among the three groups (*p* < 0.001) (Table 1). The concentration of HBP [11.05 (8.01–16.12) ng/mL] in the viral group was significantly lower than that in the bacterial group [48.85 (33.57–78.54) ng/mL] (*U* = 258.5, *p* < 0.001) and fungal group [52.90 (8.51–126.00) ng/mL] (*U* = 103.0, *p* < 0.001). However, there was no significant difference in the concentration of HBP between the bacterial and fungal groups (Fig. 1A). Because HBP is released by neutrophils, we tested the correlation between concentration of HBP and numbers of neutrophils, and found a high correlation between the two biomarkers (r_s_ = 0.421, *p* < 0.001).

#### CRP

The concentration of CRP in the bacterial and fungal groups was higher than that in the viral group; however, there were no significant differences in the concentration of CRP among the three groups (Table 1, Fig. 1B).

#### PCT

There were significant differences in the concentration of PCT among the three groups (*p* < 0.001) (Table 1). The concentration of PCT [0.17 (0.06–0.43) ng/mL] in the viral group was significantly lower than that in the bacterial group [1.0 (0.50–2.45) ng/mL] (*U* = 384.0, *p* < 0.001) and fungal group [0.80 (0.45–4.0) ng/mL] (*U* = 81.0, *p* < 0.001). There was no significant difference in concentration of PCT between the bacterial and fungal groups (Fig. 1C).

### Leukocyte and neutrophils

There were significant differences in the leukocyte and neutrophils counts among the three groups (*p* < 0.001) (Table 1). The leukocyte counts [3.89 (2.13–7.64) × 10^9^/L] in the viral group were significantly lower than those in the bacterial group [8.99 (6.45–13.24) × 10^9^/L] (*U* = 598.5, *p* < 0.001) and fungal group [9.38 (5.78–14.62) × 10^9^/L] (*U* = 114, *p* < 0.001). The leukocyte count in the bacterial group did not differ significantly from that in the fungal group. The neutrophil count in the viral group was significantly lower than that in the bacterial and fungal groups, but the count in the bacterial group did not differ significantly from that in the fungal group (Figs. 1D and 1E).

### HBP and PCT concentrations distinguished bacterial and fungal from viral infections

**Figure 2:**
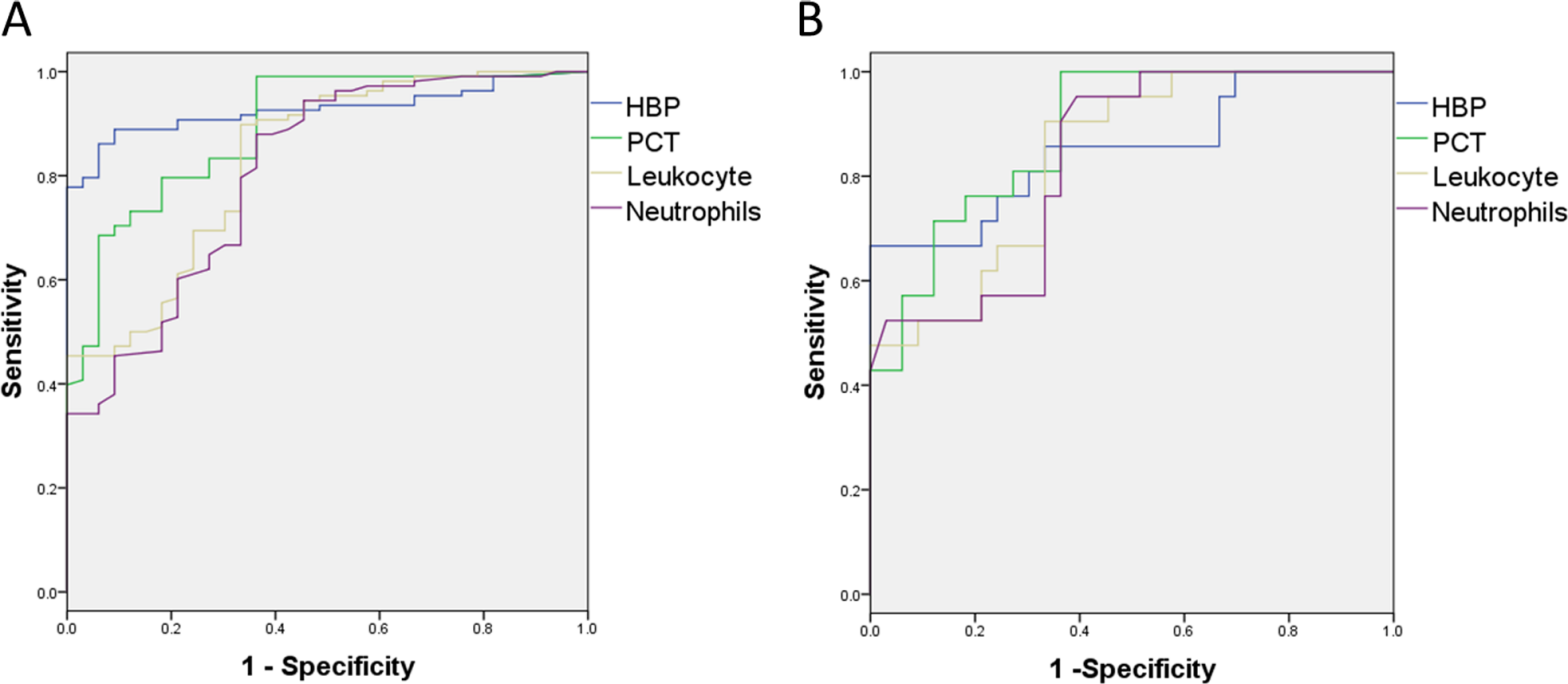
The sensitivity and specificity of inflammatory markers for distinguishing bacterial infection and fungal infection from viral infection. (A) The AUC of HBP, PCT, leukocyte and neutrophils counts for distinguishing bacterial infection from virus infection are 0.927, 0.892, 0.832 and 0.806, respectively. (B) The AUC of HBP, PCT, leukocyte and neutrophils counts for distinguishing fungal infection from virus infection are 0.851, 0.883, 0.835 and 0.830, respectively.

We found significant differences in the concentrations of HBP and PCT and leukocyte and neutrophils counts between the bacterial and viral groups. The areas under the ROC curve (AUCs) for concentration of HBP and PCT, and leukocyte as well as neutrophils counts for distinguishing bacterial infection from viral infection were 0.927, 0.892, 0.832 and 0.806 , respectively (Fig. 2A). The best cut-off value was 20.050 ng/mL for HBP, with a sensitivity of 0.861 and a specificity of 0.939. The positive predictive value (PPV) was 0.979 and negative predictive value (NPV) was 0.674, and the positive likelihood ratio (PLR) was 14.208 and negative likelihood ratio (NLR) was 0.148 for discrimination of bacterial from viral infection (Table 3). The sensitivity and specificity of PCT for distinguishing bacterial from viral infection were similar to those with HBP.

**Table 3:** The sensitivity and specificity of inflammatory markers for distinguishing bacterial and fungal infection from viral infection.

Indicators		Maximum Youden index	The best cut-off point	Sensitivity	Specificity	PPV	NPV	PLR	NLR
bacteria VS virus	HBP	0.801	20.050ng/mL	0.861	0.939	0.979	0.674	14.208	0.148
	PCT	0.627	0.195ng/mL	0.991	0.636	0.843	0.436	2.725	0.015
	Leukocyte	0.565	5.195 × 10^9^/L	0.694	0.667	0.872	0.400	2.694	0.153
	Neutrophils	0.516	4.000 × 10^9^/L	0.880	0.636	0.888	0.618	2.419	0.189
fungus VS virus	HBP	0.636	29.950ng/mL	0.667	0.970	0.933	0.821	22.000	0.344
	PCT	0.593	0.560ng/mL	0.714	0.879	0.789	0.829	5.893	0.325
	Leukocyte	0.571	5.265 × 10^9^/L	0.905	0.667	0.633	0.880	2.714	0.143
	Neutrophils	0.558	3.850 × 10^9^/L	0.952	0.606	0.606	0.952	2.418	0.079

**Notes.**

HBP heparin binding protein PCT procalcitonin PPV positive predictive value NPV negative predictive value PLR positive likelihood ratio NLR negative likelihood ratio

The AUCs of concentration of HBP and PCT, and leukocyte and neutrophils counts for distinguishing fungal infection from viral infection were 0.851, 0.883, 0.835 and 0.830, respectively (Fig. 2B). The best cut-off value was 29.950 ng/mL for HBP, with a sensitivity of 0.667 and a specificity of 0.970, PPV 0.933 and NPV 0.821, and PLR 22.0 and NLR 0.344 for discrimination of fungal from viral infection. The best cut-off value was 0.560 ng/mL for PCT, with a sensitivity of 0.714 and specificity of 0.879, PPV 0.789 and NPV 0.829, and PLR 5.893 and NLR 0.325 for discrimination of fungal from viral infection (Table 3).

## Discussion

We studied the performance of inflammatory indicators in CAP caused by bacteria, fungi and viruses. The concentration of HBP and PCT and leukocyte and neutrophils counts were significantly higher in the bacterial and fungal groups compared with the viral group.

Our data showed that HBP concentration in bacterial and fungal infections was significantly higher than that in the viral pneumonia group. Our results confirmed previous research showing that the concentration of HBP was significantly increased in bacterial compared with non-bacterial infection (Chalupa et al., 2011; Kandil et al., 2018). This differed from a previous study that showed that HBP concentration was not significantly different in bacterial compared with viral infection (Havelka et al., 2020). This might be because HBP was analyzed in patients with viral respiratory infections but not viral pneumonia, and patients with bacterial pneumonia. HBP is a granular protein synthesized during maturation in the bone marrow (Jenne, 1994) and is released by neutrophils at the site of inflammation (Soehnlein & Lindbom, 2009). We speculate that bacteria and fungi could stimulate synthesis and secretion of HBP by neutrophils. The synthesis and release of HBP had been reported in several bacterial infections. For example, The mechanism of the release of HBP might be induced by suilysin of Streptococcus that was caused by a calcium influx-dependent degranulation (Chen et al., 2016), or by M1 protein–fibrinogen complexes from *Streptococcus*, or by *Staphylococcus aureus* virulence factor phenol-soluble modulin *α*4 required formyl peptide receptor 2 and phosphoinositide 3-kinase and depended on Ca^2+^ influx and cytoskeleton rearrangement (Li et al., 2016). Research on the mechanism of induction of HBP release by fungi is still lacking. We showed that the concentration of HBP was significantly positively correlated with neutrophils count, and we speculate that more-stimulated neutrophils release more HBP.

In addition, HBP had higher predictive value for differentiating bacterial and viral infections than leukocyte and neutrophils counts. A previous study showed the best cut-off value of concentration of HBP was 45.3 ng/mL (Kandil et al., 2018), which is higher than our result of 20.05 ng/mL. So it was more representative in identifying bacterial infection and viral infection in patients. Similarly, HBP concentration was significantly increased in fungal compared with viral infection. Although there has been a lack of research on the mechanism of HBP induction by fungi, HBP is valuable for distinguishing fungal and viral CAP. Our study suggested that HBP could be used to identify bacterial, fungal and viral infection. This is helpful for treatment decision-making by clinicians.

Our data showed that the concentration of PCT was significantly higher in bacterial and fungal compared with viral infections, but there was no significant difference between bacterial and fungal infections, and PCT was helpful for distinguishing bacterial and fungal infections from viral infection in patients with CAP. Similarly, in a previous study, serum PCT levels were higher in bacterial compared with viral CAP (Bello et al., 2014), but another study showed that PCT levels were higher in bacterial infection than viral and fungal infections (Tang, Gao & Zou, 2018). Different from our research, several studies have shown that PCT had limited value in identifying bacterial and viral infections (Don et al., 2009; Hoffmann et al., 2001; Kawamatawong, Apiwattanaporn & Siricharoonwong, 2017). Synthesis of PCT is elevated by many factors, such as the non-infectious systemic inflammatory response syndrome (Aouifi et al., 1999). Bacterial and fungal pneumonia promotes more PCT secretion than viral pneumonia does. Therefore, we consider that the concentration of PCT is valuable for distinguishing viral from non-viral CAP.

There were several limitations to our study. Firstly, we only recruited patients with pneumonia and did not include those with infection of other regions. Secondly, the numbers of cases in the viral and fungal infection groups were small. Thirdly, most of our patients had complications that might have affected the level of biomarkers; for example, expression of HBP is higher among patients with cardiovascular diseases, such as atherosclerosis, myocarditis, myocardial infarction and myocardial ischemia than in normal hearts (Cai et al., 2020). The PCT levels are significantly higher in acute ischemic stroke patients as compared with normal controls (Deng et al., 2015; Marinovic et al., 2019; Yan et al., 2020). We found no significant differences in the proportion of complications, except tumor, among the different groups. Thus, we speculate that the complications had little effect on our results.

## Conclusions

Our results indicated that the plasma concentration of HBP and PCT was lower in viral than bacterial and fungal CAP. The stronger immune response characterized by higher inflammation markers including HBP and PCT can help distinguish bacterial, fungal and viral CAP.

##  Supplemental Information

10.7717/peerj.11056/supp-1 Data S1 Raw data

10.7717/peerj.11056/supp-2 File S1 Code

## Additional Information and Declarations

### Data Availability

The following information was supplied regarding data availability: Raw data and code are available in the Supplemental Files.
