# Supplementary material for: Heparin-binding protein and procalcitonin in the diagnosis of pathogens causing community-acquired pneumonia in adult patients: a retrospective study
Source: PeerJ. 2021 Mar 12;9:e11056. doi: 10.7717/peerj.11056 (PMC7958890; doi:10.7717/peerj.11056)
Supplement: File S1 [file peerj-09-11056-s002.docx]

HBP: heparin-binding protein

CRP: C-reactive protein

PCT: procalcitonin

HBV: hepatitis B virus

HCV: hepatitis C virus

HIV: human immunodeficiency virus

ALT: alanine aminotransferase

TB: total bilirubin

BUN: blood urea nitrogen

Cr: creatinine

OI: Oxygenation index

PaO_2_: partial pressure of oxygen

FiO_2_：Fraction of inspiration oxygen
